# Serum metabolomic profile of hair dye use

**DOI:** 10.1038/s41598-023-30590-3

**Published:** 2023-03-07

**Authors:** Jung-eun Lim, Jiaqi Huang, Stephanie J. Weinstein, Dominick Parisi, Satu Mӓnnistö, Demetrius Albanes

**Affiliations:** 1 Metabolic Epidemiology Branch, Division of Cancer Epidemiology and Genetics, National Cancer Institute, National Institutes of Health, 9609 Medical Center Drive, Bethesda, MD 20892 USA; 2 National Clinical Research Center for Metabolic Diseases, Key Laboratory of Diabetes Immunology, Ministry of Education, and Department of Metabolism and Endocrinology, The Second Xiangya Hospital of Central South University, Changsha, 410011 Hunan China; 3 Information Management Services, Calverton, MD USA; 4 Department of Public Health and Welfare, Finnish Institute for Health and Welfare, Helsinki, Finland

**Keywords:** Molecular biology, Biomarkers

## Abstract

The International Agency for Research on Cancer reported that some chemicals in hair dyes are probably carcinogenic to those exposed to them occupationally. Biological mechanisms through which hair dye use may be related to human metabolism and cancer risk are not well-established. We conducted the first serum metabolomic examination comparing hair dye users and nonusers in the Alpha-Tocopherol, Beta-Carotene Cancer Prevention Study. Metabolite assays were conducted using ultrahigh performance liquid chromatography-tandem mass spectrometry. The association between metabolite levels and hair dye use was estimated using linear regression, adjusting for age, body mass index, smoking, and multiple comparisons. Among the 1,401 detected metabolites, 11 compounds differed significantly between the two groups, including four amino acids and three xenobiotics. Redox-related glutathione metabolism was heavily represented, with *L*-cysteinylglycine disulfide showing the strongest association with hair dye (effect size (β) =  −  0.263; FDR adjusted *p*-value = 0.0311), along with cysteineglutathione disulfide (β =  − 0.685; FDR adjusted *p*-value = 0.0312). 5alpha-Androstan-3alpha,17beta-diol disulfate was reduced in hair dye users (β =  − 0.492; FDR adjusted *p*-value = 0.077). Several compounds related to antioxidation/ROS and other pathways differed significantly between hair dye users and nonusers, including metabolites previously associated with prostate cancer. Our findings suggest possible biological mechanisms through which the use of hair dye could be associated with human metabolism and cancer risk.

## Introduction

Hair dye products represent one of the most rapidly growing beauty and personal care industries worldwide^1^. The global market for hair coloring reached over 29 billion U.S. dollars in 2019 and is expected to increase beyond 40 billion U.S. dollars by 2023^2^. Dyeing hair involves its treatment with various natural (and/)or artificial chemical compounds mainly for cosmetic purposes (i.e., compensating for hair pigmentation loss or for altering hair color).

The association between hair dye application and the development of cancer has been an area of research during the past several decades. Our recent prospective analysis with a 28-year period of observation found that men who used hair dye experienced substantially higher prostate cancer risk than men who did not^3^. The finding is supported by a hospital-based case–control study reporting a positive association between hair dye use and prostate cancer^4^ and a cohort analysis of hairdressers which showed increased prostate cancer incidence^5^. Regarding other cancers, a Danish linkage study of census occupation and cancer register data that found both male and female hairdressers having elevated risk for bladder cancer^6^, with data from similar linkages in Finland, Norway, and Sweden showing increased risks for both bladder and lung cancer among male hairdressers^7^, who in another were found to have excess incidence of oropharyngeal and prostate cancer during 1970–80^5^. A previous meta-analysis of cancer risk among hairdressers confirmed an overall higher cancer risk compared to the general population^8^.

The International Agency for Research on Cancer (IARC) reported that some of the chemicals in hair dyes are probably carcinogenic to those who are exposed to them occupationally (e.g., hairdressers and barbers)^9^. Some of the chemical components used in hair dye products, including 4-aminobiphenyl, 1,4-phenylenediamine (PPD), 2-naphthylamine, ortho-toluidine, 2-hydroxy-1,4-naphthoquinone, and *p*-toluylenediamine, were reported to be carcinogenic in animals^9,10^. CYP-mediated N-hydroxylamine formation is regarded as a key step in the activation of aromatic amines that are known human bladder carcinogens (e.g., 4-aminobiphenyl, 2-naphthylamine, benzidine). In addition, N-acetylation is a well-established pathway for metabolism of aromatic amines^11^. Metabolic studies of the most widely used hair dye aromatic amines show they are N-acetylated in the skin by N-acetyltransferase 1 (NAT1), and N-acetylation of three aromatic amines (PPD, 2,5-diaminotoluene, and 4-amino-2-hydroxytoluene), all widely used as precursors in oxidative hair dye formulations, reduces their genotoxic potential^12^. In addition, a recent experimental study which investigated the effects of hair color changing agents on oxidative status in serum and liver specimens of rats showed permanent hair dyes had oxidative and hepatotoxic effects beyond the changing hair color^13^. Despite such evidence,, the association of personal hair dye use and cancer risk in the general population is unclear, and possible biological mechanisms through which the use of hair dye can impact human metabolism and carcinogenesis have not been firmly established.

Metabolomic examination of biologic samples is a relatively new laboratory analytical tool that identifies and measures a broad array of low-molecular-weight biochemicals in a variety of matrices including blood, urine, or organ-specific tissue, among others^14^. To our knowledge, there are no published metabolomic studies of the biochemical response to hair dye use or metabolomic profiles of hair dye users. In the present study, we compared the serum metabolomic profiles of male hair dye users and nonusers in the Alpha-Tocopherol, Beta-Carotene Cancer Prevention (ATBC) Study to identify metabolite differences related to biological mechanisms relevant to how hair dye use may influence human health.

## Results

Baseline characteristics of the study participants are presented in Table 1. Educational level and serum α-tocopherol were significantly higher in hair dye users compared with nonusers, with other factors showing no differences.

**Table 1:** Participant baseline characteristics by hair dye use in the ATBC Study^a^.

	Hair dye nonusers (N = 125)	Hair dye users (N = 75)	*p*-value
Age (years)	59.0 ± 5.0	59.0 ± 4.9	0.90
Body mass index (kg/m^2^)	26.0 ± 3.7	26.2 ± 3.1	0.69
Cigarettes/day	19.3 ± 8.7	18.6 ± 8.2	0.59
Smoking duration (years)	37.0 ± 9.7	36.4 ± 10.2	0.68
Education/training above 8th grade (%)	59.2	74.7	0.04
Alcohol (ethanol g/day)	15.4 ± 18.0	17.3 ± 15.6	0.45
Serum chemistry			
Total cholesterol (mmol/L)	6.17 ± 1.2	6.11 ± 1.2	0.73
α-Tocopherol (mg/L)	11.6 ± 3.1	12.6 ± 3.4	0.03
β-Carotene (ug/L)	216 ± 162	242 ± 244	0.41
Retinol (ug/L)	563 ± 124	594 ± 141	0.10
Trial α-tocopherol supplementation (%)	49.6	46.7	0.80
Trial β-carotene supplementation (%)	53.6	49.3	0.66

^a^Mean ± standard deviation (all values except as noted).

Among the 1,401 detected metabolites, 117 known compounds were associated with hair dye use at the nominal *p*-value < 0.05 level of statistical significance after adjustment for age, body mass index (BMI), and cigarettes smoked daily. Following additional adjustment for multiple comparisons (FDR < 0.1), 11 compounds, including four amino acids, two lipids (5alpha-androstan-3alpha,17beta-diol disulfate and O-phosphoethanolamine), one nucleotide (3-methylcytidine**)**, one peptide (fibrinopeptide B (1–11)), and three xenobiotics including nornicotine and 2,4-dihydroxyacetophenone 5-sulfate, differed significantly between the two groups (Table 2 and Table S1). Redox-related glutathione (GSH) metabolism was heavily represented (three metabolites), with L-cysteinylglycine disulfide showing the strongest association with hair dye use (β =  − 0.263), along with cysteineglutathione disulfide (β =  − 0.685), and oxidized Cys-Gly (β =  − 0.325); FDR-adjusted *p*-values of 0.0311, 0.0312, and 0.0312, respectively. N-acetyl-L-methionine was also inversely associated with hair dye use (β =  − 0.244; FDR-adjusted *p*-value of 0.036).

**Table 2:** Metabolites associated with hair dye use in the ATBC Study at FDR adjusted *p*-value < 0.1^a^.

Chemical class and metabolite	Chemical sub-class and biochemical pathway	Effect size (β)^b,c^	*p*-value	FDR adjusted *p*-value
Amino acids				
L-Cysteinylglycine disulfide	Glutathione metabolism	− 0.263	< 0.0001	0.0311
Cysteineglutathione disulfide	Glutathione metabolism	− 0.685	< 0.0001	0.0312
Cys-Gly, oxidized	Glutathione metabolism	− 0.325	< 0.0001	0.0312
N-Acetyl-L-methionine	Methionine, cysteine, SAM and taurine metabolism	− 0.244	0.0001	0.036
Lipids				
5alpha-Androstan-3alpha,17beta-diol disulfate	Androgenic steroids	− 0.492	0.0008	0.077
O-Phosphoethanolamine	Phospholipid metabolism	− 0.316	0.0008	0.077
Nucleotides				
3-Methylcytidine	Pyrimidine metabolism, cytidine containing	− 0.173	0.0004	0.058
Peptides				
Fibrinopeptide B (1–11)	Fibrinogen cleavage peptide	− 0.308	0.0005	0.061
Xenobiotics				
2,4-Dihydroxyacetophenone 5-sulfate	Benzoate metabolism	0.863	0.0004	0.058
Umbelliferone sulfate	Food component/plant	0.958	< 0.0001	0.0312
Nornicotine	Tobacco metabolites	− 0.382	0.0002	0.036

^a^*ATBC* Alpha-Tocopherol, Beta-Carotene Cancer Prevention; *FDR* false discovery rate.

^b^The effect sizes and *p* values were estimated using linear regression.

^c^Adjusted for age, body mass index, and number of cigarettes smoked daily.

Table S1 presents all metabolites associated with hair dye exposure at *p*-value < 0.05 including those presented in Table 2. Twenty-two amino acid metabolites including thyroxine were inversely associated with hair dye use, along with 59 lipids including sphingomyelins, nine nucleotides (e.g., deoxyuridine), and seven peptides (e.g., gamma-glutamylserine). Among 10 associated xenobiotics, six were positively associated with hair dye use, with the strongest signal for umbelliferone sulfate (β = 0.958; *p*-value < 0.0001); sphingomyelins and several tricarboxylic acid (TCA) cycle-related metabolites were also associated with hair dye use.

In the gene set analysis (GSA), no metabolite chemical sub-pathway exceeded the FDR adjusted *p*-value threshold of 0.01. Sphingomyelins and gamma-glutamyl amino acids were inversely associated with hair dye use at the nominal *p*-value < 0.05 level of statistical significance (*p* = 0.01 and *p* = 0.024, respectively; data not shown).

**Figure 1:**
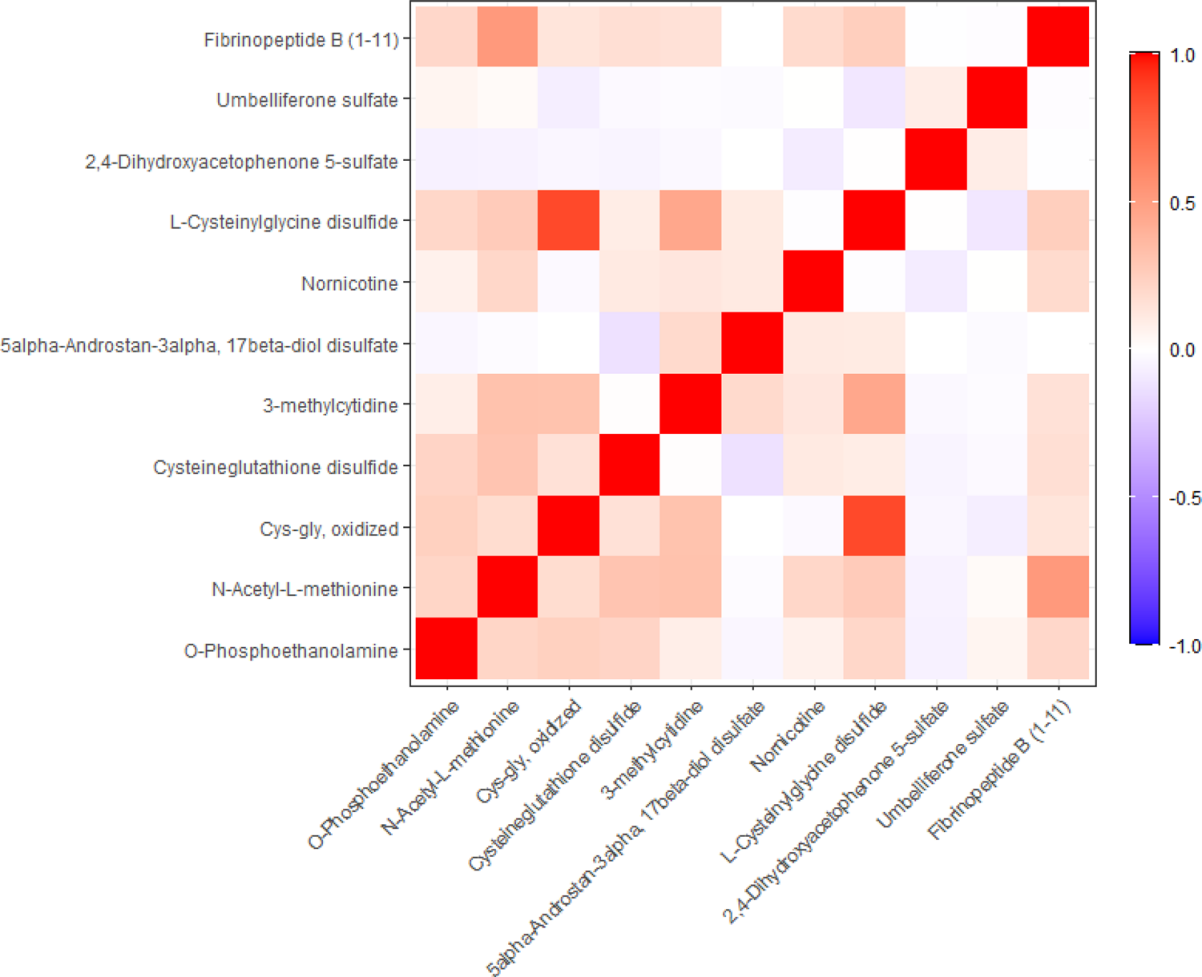
Heat map of correlation coefficients for metabolites that differed significantly between hair dye users and nonusers at FDR-adjusted *p*-value < 0.1.

Figure 1 depicts a heat map of correlations among metabolites that differed between hair dye users and nonusers at the FDR-adjusted *p*-value of < 0.1. With the exception of oxidized Cys-Gly and L-cysteinylglycine disulfide (r = 0.87), only weak to modest correlations were observed among the metabolites.

## Discussion

In this first serum metabolomic examination of hair dye use, we identified 11 metabolites which differed between men who used hair dyes and those who did not, including three metabolites related to GSH redox metabolism. Several of these metabolites may reflect both response to hair dye exposure and potential biological mechanisms for their association with health outcomes, including most recently prostate cancer as reported in the ATBC Study^15^.

Reduced GSH-related metabolites in hair dye users, including L-cysteinylglycine disulfide, cysteineglutathione disulfide, and oxidized Cys-Gly could indicate metabolism of increased production of oxidative stress-related reactive oxygen species (ROS). GSH is a small, low molecular weight, water-soluble thiol-tripeptide, synthesized from glutamate, cysteine and glycine, which serves as a key intracellular antitoxicant in the metabolism of endogenous and xenobiotic compounds through conjugation and excretion^16,17^. Gamma-glutamyl amino acids are key intermediates of the gamma-glutamyl cycle which helps maintain intracellular GSH levels and are critical to the cellular response to oxidative stress^18^. The gamma-glutamyl transferase 1 (GGT1) is a membrane transpeptidase that removes the gamma-glutamyl moiety from extracellular GSH, oxidized GSH, or GSH conjugates and transfers it to an amino acid acceptor. The observed association between the gamma-glutamyl amino acids chemical sub-pathway and hair dye use also supports the influence of hair dye use on oxidative stress. Alternatively, the finding may reflect reduced glutathione production (or excretion to the extracellular compartment) in hair dye users. Our study was purposefully conducted as an untargeted, “broad-spectrum” metabolomics analysis for the purposes of hypothesis-generation related to the biochemical, biological and health effects of exposure to hair dye. Although detectable by the platform utilized, GSH and GSSG appear to have not been present in sufficient concentrations in our study samples even though we observed signals for related species. Our findings provide hypothesis-testing opportunities for similar future studies to examine GSH metabolism and gamma-glutamyl amino acid-derived metabolites through targeted metabolomic profiling. Because of the dynamic nature of thiol-disulfide exchange reactions and methodologic artifacts that can interfere with their measurement, further methodological developments for the measurement of thiols and disulfides would be helpful.

Melanocyte pigment production gives hair its color, and intracellular antioxidant defense mechanisms play a vital role in maintaining an optimal redox balance by quenching ROS^19^. Also, melanin (as well as GSH) production declines with age, which in turn results in hair greyness, and people with more gray hair and reduced GSH production may have been more likely to use hair dyes. Although we were unable to compare the degree of hair greyness between hair dye users and non-users based on the original mutually exclusive question regarding hair, we did strictly adjust for age in all analyses. Our finding for N-acetyl-*L*-methionine may also be related to increased oxidative stress given that methionine is one of the more readily oxidized residues in proteins which can be attacked by many ROS in vivo^20^.

Perhaps most important regarding findings from the present investigation is that several compounds associated with prostate cancer in our previous etiologic studies were significantly associated with hair dye use. For example, the prostate cancer serum metabolomic risk profile showed associations for lipid, TCA cycle, and amino acid metabolites^21,22^, with the lower lysolipids, inositols, and sphingomyelins implicating cell membrane biosynthesis and intracellular signaling^23^. Although the main purpose of this study is to identify metabolite differences related to hair dye use rather than cancer risk, considering the positive association between hair dye use and prostate cancer risk we observed in our recent prospective analysis which substantiated a prior hospital-based case–control study and identified significant associations for sphingomyelin, fibrinopeptide, androgenic, and TCA cycle-related metabolites^3,4^, the present findings point to possible biological mechanisms underlying the hair dye risk association, including 5alpha-androstane-3beta,17beta-diol which we found to be reduced in hair dye users and which inhibits prostate cancer cell migration through ER_β_ activation^24^.

3-Methylcytidine has been investigated as a potential biomarker for cancer and has been found to be significantly elevated in the urine of breast cancer patients^25^. Although the relationship between serum 3-methylcytidine and urinary 3-methylcytidine is not established, significant difference of serum 3-methylcytidine level in hair dye users may reflect the potential carcinogenic properties of hair dyes. Fibrin and fibrin degradation products including fibrinopeptides activate and regulate inflammatory responses to injury. Fibrinopeptide B is a known potent chemoattractant, and its preferential release suggests a physiological purpose in cellular response to injury^26^. Tissue damage induced by permanent hair dyes has been reported^27^, suggesting that the observed fibrinopeptide B association may reflect such inflammatory responses.

Umbelliferone, a natural product of the coumarin family, was also increased in hair dye users. Coumarin was classified as a toxic substance by the U.S. Food and Drug Administration (FDA) in 1954 after it was found to induce liver tumorigenesis in rats^28^, and the consumption of all foods containing coumarins was subsequently prohibited. Also, the National Institute for Occupational Safety and Health has named coumarin as a chemical carcinogen based on additional rodent experiments^28^, and the use of B-methylumbelliferone as color additives for cosmetic products is also no longer authorized by the FDA^29^. If some hair dye users were exposed to such hair color additives before these restrictions were implemented, it could have led to elevated serum umbelliferone. Alternatively, greater use of other preparations containing umbelliferone, including ultraviolet B-blocking sun lotions, could be responsible for the difference.

We also found nornicotine was reduced among hair dye users. An experimental study showed that hair treatments including dyes, permanent wave, and hydrogen peroxide lead to substantially decreased hair nicotine content in smokers, although the effects of the various treatments were not reproducible^30^. As a result, the depletions were explained as probably being due to degradation of the compounds rather than to a leaching effect. 2,4-Dihydroxyacetophenone 5-sulfate is another xenobiotic that was positively associated with hair dye use in our investigation. The metabolite belongs to the alkyl-phenylketone class of plant-based aromatic organic compounds, and people are mainly exposed to this substance through diet and herbal remedies.

Our study has several notable strengths. It is the first metabolomic examination of hair dye exposure, and it was conducted in a large, well-characterized cohort. We assayed fasting serum which reduced biochemical variation related to recent food and beverage consumption, either directly (i.e., through increased ingested metabolites) or indirectly (i.e., through up-regulated or down-regulated metabolism of similar nutritional compounds), thereby offering snapshots of “basal” metabolomic profiles of hair dye exposure. Importantly, we were able to identify more than 1,400 metabolites representing a wide range of biochemical pathways. Limitations of the study include that metabolites were measured at only one timepoint based on the parent study design. Hair dye use was ascertained through a self-reported questionnaire without repeated ascertainment of the exposure during follow-up or information regarding frequency of use and specific hair dye chemical formulations. The precise hair dye formulation can change over time. The homogenous nature of our male smoker population of European ancestry may limit generalizability of our findings to other populations. The time interval between the most recent hair dye usage and collection of study serum was not collected. Although the oxidative effects of hair dye may decline with time after application, it is unlikely (but possible) that the hair dye was used just before blood sampling (i.e., within a few hours). Other unmeasured or residual confounding remains possible even though we adjusted for several potential confounding factors. Future studies should aim for larger sample sizes in order to improve reliability of metabolites measurements and take possible seasonal variation into consideration when being designed.

In conclusion, in this first metabolomic investigation of human exposure to hair dyes, we identified several metabolites having plausible biological mechanisms through which hair dye impacts metabolism and may influence prostate cancer risk. Our findings provide hypothesis-testing opportunities for similar future studies to examine GSH metabolism and gamma-glutamyl amino acid-derived metabolites through targeted assays. Our findings also warrant re-examination in other, more diverse study populations with hair dye information, including frequency and duration of use, as well as usual brands or chemical compositions.

## Methods

### Study population

Study methods of the ATBC Study have been described^31^. Male Finnish smokers (*N* = 29,133) ages 50–69 years were assigned to receive either alpha-tocopherol (*dl*-α-tocopheryl-acetate, 50 mg/day), beta-carotene (20 mg/day), both vitamins, or placebo for 5–8 years (a median of 6.1 years). At enrollment, participants completed questionnaires regarding behavioral and lifestyle information, including hair greyness and hair dye use, smoking intensity, smoking duration, and education level. BMI was calculated based on measured weight (in kilograms) divided by height (in meters squared). The use of hair dye was identified in one question that also asked about hair greyness and balding. The response categories were: no grey hair; less than 25%, about 25%, about 50%, about 75%, almost all grey, or all grey hair; bald; or dyed hair. The current analysis included all the hair dye users (*N* = 75) and 125 controls who did not report using hair dye who were frequency-matched to hair dye users based on age (± 6 months). Fasting serum was collected at baseline and stored at − 80 °C until assayed for this study. Total cholesterol concentrations were measured following enrollment using an enzymatic assay, and serum alpha-tocopherol, beta-carotene, and retinol were assayed using high-performance liquid chromatography in one laboratory.

The ATBC Study was approved by institutional review boards at both the U.S. National Cancer Institute and the Finnish National Public Health Institute, and written informed consent was obtained from all participants. All methods were performed in accordance with the relevant guidelines and regulations.

### Metabolomic analysis

Serum metabolomic examination was conducted at Metabolon, Inc. (Durham, N.C.) according to previously described methods^32^. All methods utilized a Waters ACQUITY ultra-high-performance liquid chromatography (UPLC) and a Thermo Scientific Q-Exactive high resolution/accurate mass spectrometer interfaced with a heated electrospray ionization (HESI-II) source and Orbitrap mass analyzer operated at 35,000 mass resolution. The method details of sample preparation, data extraction, compound identification, and quality assurance/quality control have also been described before^32–35^. Methodological details of the metabolomic analyses are provided in collaboration with Metabolon. Briefly, samples were prepared using the automated MicroLab STAR® system from Hamilton, Inc. The sample extract was dried then reconstituted in solvents compatible to each of the four platform assay methods using ultrahigh performance liquid chromatography-tandem mass spectrometry (MS). Each reconstitution solvent contained a series of standards at fixed concentrations to ensure injection and chromatographic consistency. One aliquot was analyzed using acidic positive ion conditions, chromatographically optimized for more hydrophilic compounds. In this method, the extract was gradient-eluted from a C18 column (Waters UPLC BEH C18-2.1 × 100 mm, 1.7 µm) using water and methanol, containing 0.05% perfluoropentanoic acid (PFPA) and 0.1% formic acid. Another aliquot was also analyzed using acidic positive ion conditions but was chromatographically optimized for hydrophobic compounds. In this method, the extract was gradient-eluted from the same aforementioned C18 column using methanol, acetonitrile, water, 0.05% PFPA and 0.01% formic acid and was operated at an overall higher organic content. A third aliquot was analyzed using basic negative ion-optimized conditions using a separate dedicated C18 column. The basic extracts were gradient-eluted from the column using methanol and water with 6.5 mM ammonium bicarbonate at pH 8. The fourth aliquot was analyzed via negative ionization following elution from a HILIC column (Waters UPLC BEH Amide 2.1 × 150 mm, 1.7 µm) using a gradient consisting of water and acetonitrile with 10 mM ammonium formate, pH 10.8. The MS analysis alternated between MS and data-dependent MSn scans using dynamic exclusion.

The raw data were further extracted and peak-identified using Metabolon’s hardware and software^32,33,35^. MS2 spectra were acquired and reviewed as part of the biochemical curation process. For the identification of molecules, Metabolon matches ion chromatographic retention index, accurate mass and mass spectral fragmentation signature with a reference library of authentic standards. Molecules are Tier 1 identification unless they are including an * or ** after the biochemical name. After excluding metabolites that were missing for more than 90% of the 200 study samples, 1,339 identified compounds remained eligible for analysis among the 1,401 detected metabolites.

Based upon standard chemical classification, a general biochemical class was assigned to the identified biochemicals (e.g., lipid, amino acid, carbohydrate, cofactor and vitamin, energy metabolite, nucleotide, peptide, and xenobiotic). More specific chemical subclasses and pathways were also attributed to each known metabolite (e.g., GSH metabolism, benzoate metabolism, etc.).

To evaluate the technical reliability of the assay, two blinded quality control (QC) samples from a pooled serum sample were included in each batch. The median and interquartile range of the coefficient of variation (CV) and intraclass correlation coefficient (ICC) across the metabolites were 8% (4%-14%) and 0.98 (0.96–0.99), respectively. Several other internal controls were analyzed in concert with the experimental serum samples. These included 1) a pooled matrix sample generated by taking a small volume of each experimental sample (or alternatively, use of a pool of well-characterized human serum) that served as a technical replicate throughout the sample batches, 2) extracted water samples serving as processing blanks, and 3) and a cocktail of QC standards that were carefully chosen not to interfere with the measurement of endogenous compounds and were spiked into every analyzed sample which permitted monitoring of instrument performance and aided chromatographic alignment. Values for instrument and process variability met acceptance criteria with a 5% median relative standard deviation (RSD) for internal standard QC samples, and 10% median RSD for endogenous biochemical QC samples.

### Statistical analysis

To account for batch variability, metabolite values were divided by their batch median value, log-transformed and centered according to normalization. For each metabolite, the minimum value across all batches is imputed for the metabolite values below the limit of detection^33,34^.

The association between hair dye exposure and each metabolite was examined using linear regression, adjusting for age at randomization, BMI, and number of cigarettes smoked daily (all as continuous variables). To control for multiple comparisons, we applied a false discovery rate (FDR) correction using *p* = 0. 1 as the adjusted significance threshold.

We also conducted biochemical pathway GSA to evaluate whether pre-defined metabolic sub-pathways differed between hair dye users and nonusers^36^. The GSA R package was used with 10,000 permutations.

All the analyses were performed using SAS statistical software version 9.4 (SAS Institute, Cary, NC) and R 3.6.3. All reported *p*-values were two-sided.

## Supplementary Information

Supplementary Information.

## Data Availability

The data analyzed for this study are available from the corresponding author upon reasonable request.

## Abbreviations

ATBC: Alpha-Tocopherol, Beta-Carotene Cancer Prevention
BMI: Body mass index
CV: Coefficient of variation
FDA: Food and Drug Administration
FDR: False discovery rate
GGT1: Gamma-glutamyl transferase 1
GSA: Gene set analysis
GSH: Glutathione
IARC: International Agency for Research on Cancer
ICC: Intraclass correlation coefficient
QC: Quality control
ROS: Reactive oxygen species
RSD: Relative standard deviation
TCA: Tricarboxylic acid
