# Supplementary material for: Serum metabolomic profile of hair dye use
Source: Sci Rep. 2023 Mar 7;13:3776. doi: 10.1038/s41598-023-30590-3 (PMC9992367; doi:10.1038/s41598-023-30590-3)
Supplement: Supplementary file 1 — Supplementary Information. [file 41598_2023_30590_MOESM1_ESM.docx]

**Table S1. Metabolites associated with hair dye use in the ATBC Study at P<0.05^a^**

| **Chemical class and metabolite** | **Chemical sub-class and biochemical pathway** | **Effect size (β)^b,c^** | **P-value** | **FDR adjusted P-value** |
| --- | --- | --- | --- | --- |
| **Amino Acids** |  |  |  |  |
| N-Acetyl-L-alanine | Alanine and aspartate metabolism | -0.064 | 0.020 | 0.280 |
| Creatinine | Creatine metabolism | -0.046 | 0.034 | 0.355 |
| L-Glutamine | Glutamate metabolism | -0.065 | 0.005 | 0.184 |
| L-Cysteinylglycine disulfide | Glutathione metabolism | -0.263 | <0.0001 | 0.0311 |
| Cysteineglutathione disulfide | Glutathione metabolism | -0.685 | <0.0001 | 0.0312 |
| Cys-gly, oxidized | Glutathione metabolism | -0.325 | <0.0001 | 0.0312 |
| Cysteinylglycine | Glutathione metabolism | -0.146 | 0.021 | 0.282 |
| L-Serine | Glycine, serine and threonine metabolism | -0.066 | 0.020 | 0.280 |
| Dimethylglycine | Glycine, serine and threonine metabolism | -0.079 | 0.045 | 0.420 |
| Alpha-ketoisovaleric acid | Leucine, isoleucine and valine metabolism | -0.080 | 0.023 | 0.291 |
| 3-Methylglutarylcarnitine 2 | Leucine, isoleucine and valine metabolism | -0.227 | 0.030 | 0.342 |
| Glutarylcarnitine | Lysine metabolism | -0.194 | 0.003 | 0.158 |
| N-Acetyl-L-methionine | Methionine, cysteine, SAM and taurine metabolism | -0.244 | <0.0001 | 0.036 |
| Methylcysteine | Methionine, cysteine, SAM and taurine metabolism | -0.109 | 0.014 | 0.276 |
| Taurine | Methionine, cysteine, SAM and taurine metabolism | -0.099 | 0.018 | 0.280 |
| L-Cysteine | Methionine, cysteine, SAM and taurine metabolism | -0.101 | 0.025 | 0.308 |
| Hypotaurine | Methionine, cysteine, SAM and taurine metabolism | -0.112 | 0.050 | 0.432 |
| N1-Acetylspermidine | Polyamine metabolism | -0.108 | 0.019 | 0.280 |
| C-Glycosyltryptophan | Tryptophan metabolism | -0.064 | 0.028 | 0.323 |
| Thyroxine | Tyrosine metabolism | -0.095 | 0.003 | 0.149 |
| ADMA + SDMA | Urea cycle; arginine and proline metabolism | -0.077 | 0.003 | 0.149 |
| Ornithine | Urea cycle; arginine and proline metabolism | -0.093 | 0.014 | 0.276 |
| **Carbohydrates** |  |  |  |  |
| Aspartylglycosamine | Aminosugar metabolism | -0.151 | 0.005 | 0.176 |
| N-Acetylneuraminic acid | Aminosugar metabolism | -0.085 | 0.049 | 0.429 |
| Pyruvic acid | Glycolysis, gluconeogenesis, and pyruvate metabolism | -0.221 | 0.005 | 0.179 |
| L-Lactic acid | Glycolysis, gluconeogenesis, and pyruvate metabolism | -0.091 | 0.037 | 0.376 |
| **Cofactors and Vitamins** |  |  |  |  |
| Pantothenic acid | Pantothenate and CoA metabolism | 0.110 | 0.050 | 0.432 |
| Alpha-CEHC sulfate | Tocopherol metabolism | 0.244 | 0.046 | 0.421 |
| 4-Pyridoxic acid | Vitamin B6 metabolism | 0.229 | 0.015 | 0.276 |
| **Energy** |  |  |  |  |
| Succinylcarnitine | TCA cycle | -0.107 | 0.007 | 0.191 |
| cis-Aconitic acid variant | TCA Cycle | -0.079 | 0.020 | 0.280 |
| Oxoglutaric acid | TCA cycle | -0.143 | 0.025 | 0.308 |
| **Lipids** |  |  |  |  |
| 5alpha-Androstan-3alpha,17beta-diol disulfate | Androgenic steroids | -0.492 | 0.001 | 0.077 |
| 4-Androsten-3alpha,17alpha-diol monosulfate (3) or Androstenediol (3alpha, 17alpha) monsulfate (3) | Androgenic steroids | -0.222 | 0.018 | 0.280 |
| Androsterone sulfate | Androgenic steroids | -0.261 | 0.024 | 0.300 |
| 5alpha-Androstan-3alpha,17beta-diol monosulfate (2) | Androgenic steroids | -0.264 | 0.032 | 0.347 |
| Androsterone sulfate 1 | Androgenic steroids | 0.190 | 0.045 | 0.420 |
| 16a-hydroxy DHEA 3-sulfate | Androgenic steroids | 0.200 | 0.047 | 0.423 |
| 5alpha-Androstan-3alpha,17beta-diol monosulfate (1)* | Androgenic steroids | -0.257 | 0.048 | 0.424 |
| 3b,17a-Dihydroxy-5a-androstane | Androgenic steroids | -0.198 | 0.048 | 0.428 |
| 4-Trimethylammoniobutanoic acid | Carnitine metabolism | -0.064 | 0.037 | 0.376 |
| Sphingomyelin (d18:0/14:0) | Dihydrosphingomyelins | -0.104 | 0.021 | 0.280 |
| Sphingomyelin (d18:0/16:0) | Dihydrosphingomyelins | -0.073 | 0.028 | 0.323 |
| 12S-HHT | Eicosanoids | -0.671 | 0.007 | 0.191 |
| 5-HEPE | Eicosanoids | 0.213 | 0.024 | 0.306 |
| Octadecanedioylcarnitine (C18-DC)* | Fatty acid metabolism (Acyl carnitine, dicarboxylate) | -0.216 | 0.001 | 0.111 |
| C18:1-DC Carnitine | Fatty acid metabolism (Acyl carnitine, dicarboxylate) | -0.240 | 0.002 | 0.123 |
| O-Adipoylcarnitine | Fatty acid metabolism (Acyl carnitine, dicarboxylate) | -0.181 | 0.026 | 0.316 |
| Heptadecanoyl carnitine | Fatty acid metabolism (Acyl carnitine, long chain saturated) | -0.101 | 0.038 | 0.380 |
| Stearoylcarnitine | Fatty acid metabolism (Acyl carnitine, long chain saturated) | -0.088 | 0.042 | 0.399 |
| Malonylcarnitine | Fatty acid synthesis | 0.176 | 0.006 | 0.184 |
| Octadecenedioate | Fatty acid, dicarboxylate | -0.211 | 0.002 | 0.133 |
| Octadecanedioic acid | Fatty acid, dicarboxylate | -0.163 | 0.013 | 0.276 |
| Octadecadienedioate (C18:2-DC) | Fatty acid, dicarboxylate | -0.182 | 0.030 | 0.337 |
| Hexadecanedioic acid | Fatty acid, dicarboxylate | -0.161 | 0.046 | 0.421 |
| Glycosyl-N-tricosanoyl-sphingadienine (d18:2/23:0)* | Hexosylceramides (HCER) | -0.140 | 0.016 | 0.276 |
| Glycosyl ceramide (d18:2/24:1, d18:1/24:2)* | Hexosylceramides (HCER) | -0.101 | 0.013 | 0.276 |
| Glycosyl-N-behenoyl-sphingadienine (d18:2/22:0)* | Hexosylceramides (HCER) | -0.113 | 0.021 | 0.280 |
| Galactosylceramide (d18:1/24:1(15Z)) | Hexosylceramides (HCER) | -0.208 | 0.041 | 0.392 |
| Glycosyl ceramide (d18:1/20:0, d16:1/22:0)* | Hexosylceramides (HCER) | -0.076 | 0.040 | 0.392 |
| 1-(5Z,8Z,11Z,14Z-Eicosatetraenoyl)-sn-glycero-3-phosphate | Lysophospholipids | -0.194 | 0.007 | 0.191 |
| 1-Stearoylglycerophosphoserine | Lysophospholipids | -0.357 | 0.021 | 0.280 |
| O-Phosphoethanolamine | Phospholipid metabolism | -0.316 | 0.001 | 0.077 |
| Phosphorylcholine | Phospholipid metabolism | -0.126 | 0.006 | 0.184 |
| PE(O-18:1(1Z)/20:4(5Z,8Z,11Z,14Z)) | Plasmalogens | -0.097 | 0.041 | 0.392 |
| Tauro-b-muricholic acid | Primary bile acid metabolism | 0.311 | 0.031 | 0.345 |
| Taurodeoxycholic acid | Secondary bile acid metabolism | 0.614 | 0.016 | 0.277 |
| Deoxycholic acid glycine conjugate | Secondary bile acid metabolism | 0.614 | 0.023 | 0.291 |
| Sphinganine 1-phosphate | Sphingolipid synthesis | -0.153 | 0.007 | 0.195 |
| Eicosenoyl sphingomyelin* or Sphingomyelin (d18:1/20:1, d18:2/20:0)* | Sphingomyelins | -0.122 | 0.002 | 0.123 |
| Sphingomyelin (d18:2/23:1)* | Sphingomyelins | -0.124 | 0.002 | 0.133 |
| Sphingomyelin (d18:1/18:0) | Sphingomyelins | -0.161 | 0.003 | 0.162 |
| Sphingomyelin (d18:2/21:0, d16:2/23:0)* | Sphingomyelins | -0.152 | 0.003 | 0.162 |
| Sphingomyelin (d17:1/24:1(15Z)) | Sphingomyelins | -0.104 | 0.004 | 0.162 |
| Sphingomyelin (d18:1/16:0) | Sphingomyelins | -0.060 | 0.004 | 0.162 |
| Sphingomyelin (d18:1/18:1(9Z)) | Sphingomyelins | -0.091 | 0.005 | 0.176 |
| Sphingomyelin (d18:1/18:0) | Sphingomyelins | -0.092 | 0.006 | 0.191 |
| Sphingomyelin (d17:2/16:0, d18:2/15:0)* | Sphingomyelins | -0.136 | 0.008 | 0.220 |
| Sphingomyelin (d18:1/21:0, d17:1/22:0, d16:1/23:0)* | Sphingomyelins | -0.112 | 0.008 | 0.220 |
| Palmitoleoyl sphingomyelin* or Sphingomyelin (d18:2/16:0, d18:1/16:1)* | Sphingomyelins | -0.073 | 0.008 | 0.220 |
| Sphingomyelin (d18:1/22:1(13Z)) | Sphingomyelins | -0.074 | 0.010 | 0.240 |
| Sphingomyelin (d17:1/16:0, d18:1/15:0, d16:1/17:0)* or Sphingomyelin (d18:1/15:0, d16:1/17:0)* | Sphingomyelins | -0.108 | 0.010 | 0.249 |
| Sphingomyelin (d18:1/19:0, d19:1/18:0)* | Sphingomyelins | -0.118 | 0.015 | 0.276 |
| Sphingomyelin (d18:1/17:0, d17:1/18:0, d19:1/16:0) | Sphingomyelins | -0.106 | 0.014 | 0.276 |
| Sphingomyelin (d18:2/24:2)* | Sphingomyelins | -0.096 | 0.013 | 0.276 |
| Sphingomyelin (d18:1/20:0) | Sphingomyelins | -0.073 | 0.014 | 0.276 |
| Sphingomyelin (d18:1/22:0) | Sphingomyelins | -0.102 | 0.021 | 0.280 |
| Sphingomyelin (d18:1/23:0) | Sphingomyelins | -0.084 | 0.020 | 0.280 |
| Sphingomyelin (d18:1/20:2, d18:2/20:1, d16:1/22:2)* | Sphingomyelins | -0.121 | 0.032 | 0.347 |
| Sphingomyelin (d18:1/25:0, d19:0/24:1, d20:1/23:0, d19:1/24:0)* | Sphingomyelins | -0.113 | 0.033 | 0.349 |
| Sphingosine 1-phosphate | Sphingosines | -0.118 | 0.009 | 0.235 |
| **Nucleotides** |  |  |  |  |
| Hypoxanthine | Purine metabolism, (hypo)xanthine/inosine containing | -0.104 | 0.037 | 0.376 |
| Adenine | Purine metabolism, adenine containing | -0.102 | 0.016 | 0.276 |
| 1-Methyladenosine | Purine metabolism, adenine containing | -0.065 | 0.038 | 0.380 |
| 3-Methylcytidine | Pyrimidine metabolism, cytidine containing | -0.173 | 0.000 | 0.058 |
| 2'-O-methylcytidine | Pyrimidine metabolism, cytidine containing | -0.121 | 0.027 | 0.317 |
| L-Dihydroorotic acid | Pyrimidine metabolism, orotate containing | -0.193 | 0.011 | 0.271 |
| Deoxyuridine | Pyrimidine metabolism, uracil containing | -0.128 | 0.011 | 0.260 |
| Pseudouridine | Pyrimidine metabolism, uracil containing | -0.065 | 0.015 | 0.276 |
| Dihydrouracil | Pyrimidine metabolism, uracil containing | -0.190 | 0.018 | 0.280 |
| **Peptides** |  |  |  |  |
| Phenylacetylglycine | Acetylated peptides | -0.367 | 0.013 | 0.276 |
| Fibrinopeptide B (1-11) | Fibrinogen cleavage peptides | -0.308 | 0.001 | 0.061 |
| ADpSGEGDFXAEGGGVR* | Fibrinogen cleavage peptides | -0.286 | 0.004 | 0.171 |
| Fibrinopeptide B (1-12) | Fibrinogen cleavage peptides | -0.179 | 0.026 | 0.316 |
| Gamma-Glutamylserine | Gamma-glutamyl amino acids | -0.135 | 0.006 | 0.184 |
| N,N-Dimethyl-PRO-PRO | Modified peptides | -0.085 | 0.009 | 0.230 |
| HWESASXX* | Polypeptides | 0.250 | 0.041 | 0.392 |
| **Xenobiotics** |  |  |  |  |
| 2,4-Dihydroxyacetophenone 5-sulfate | Benzoate metabolism | 0.863 | 0.000 | 0.058 |
| [2-hydroxy-6-Methoxy-4-(prop-2-en-1-yl)phenyl] Oxidanesulfonic acid | Benzoate metabolism | 0.489 | 0.016 | 0.276 |
| Salicyluric acid | Benzoate metabolism | 0.737 | 0.027 | 0.318 |
| Sulfate | Chemicals | -0.053 | 0.047 | 0.421 |
| Triethanolamine | Chemicals | 0.323 | 0.019 | 0.280 |
| Salicylic acid | Drug - topical agents | 0.776 | 0.020 | 0.280 |
| Umbelliferone sulfate | Food component/plant | 0.958 | <0.0001 | 0.031 |
| Gluconic acid | Food component/plant | -0.135 | 0.004 | 0.176 |
| 2-Keto-3-deoxy-D-gluconic acid | Food component/plant | -0.155 | 0.021 | 0.280 |
| Nornicotine | Tobacco metabolites | -0.382 | 0.000 | 0.036 |

^a^ATBC, Alpha-Tocopherol, Beta-Carotene Cancer Prevention; FDR, false discovery rate.

^b^The effect sizes and P-values were estimated using linear regression.

^c^Adjusted for age, body mass index, and number of cigarettes smoked daily
